# Comment on “Increased risk of second cancers at sites associated with HPV after a prior HPV-associated malignancy, a systematic review and meta-analysis”

**DOI:** 10.1038/s41416-019-0437-2

**Published:** 2019-03-26

**Authors:** Rama Jayaraj, Chellan Kumarasamy

**Affiliations:** 1 Clinical Sciences, College of Health and Human Sciences, Charles Darwin University, Ellengowan Drive, Casuarina, NT 0909 Australia; 2 University of Adelaide, North Terrace Campus, Adelaide, SA 5005 Australia

**Keywords:** Tumour virus infections, Infection

The systematic review and meta-analysis study “Increased risk of second cancers at sites associated with HPV after a prior HPV-associated malignancy, a systematic review and meta-analysis”, presented by Gilbert et al. and published in BJC, has been read by us with interest.^1^ Our meta-analysis research team would like to bring into attention a few valid improvements to the paper. Therefore, this study may serve a better position as a citable article.

## Small sample size and wider confidence interval for quantitative synthesis

A question that presents itself upon viewing the data presented in the study is the choice of Gilbert et al. to conduct a meta-analysis when a number of included studies have very small sample sizes. The problems that arise upon choosing to include studies with small sample sizes is self-evident in Figure 3 of Gilbert et al.’s paper, where we observed that the number of studies (the studies in question have been listed in the Table 1 given below) have very wide percent of Confidence Intervals (CI)–95% CI, going beyond the limits of the constructed Forest Plot. Wide CI’s indicate a lack of precision, and the presence of the high degree of standard error, which in turn is a characteristic symptom of a having a small sample size.^2,3^ This issue needs to be addressed as a major limitation of this study.

**Table 1:** A number of included studies have wide 95% confidence intervals (CI) in the forest plot

Author	Second primary cancer type	Number of cases
Fisher et al.	Cervical	5
Mitchell et al.	Cervical	6
Neumann et al.	Vulvo-vaginal	8
	Anal	5
	Oropharynx	2
Hemminki et al.	Cervical	7
	Anal	1
	Oropharynx	2
Saleem et al.	Anal	5
Frisch et al.	Cervical	2
	Vulvo-vaginal	5

## Individual study weight against pooled estimated effect size

Furthermore, the meta-analysis conducted does not include the mention of the individual weights of the study, which leads to further uncertainty, as readers are unable to clearly ascertain the degree of impact that the small sample size studies have upon the overall pooled Standardised Incidence Ratios (SIRs).^4^

## Is SIRs interchangeable with hazard ratio (HRs)?

The abovementioned issues are exacerbated when we observe that the authors have used the term SIRs interchangeably with HRs, particularly in the forest plots (Figure 2 and Figure 3) where they continue to describe the SIRs in the legends of each figure, while the figures themselves seem to represent HRs. The estimated or pooled effect size of the risk of HPV-associated cancer after prior diagnosis is SIR in this meta-analysis. In statistical terms, HRs are markedly different from SIRs, as the former denotes hazard rates corresponding to the conditions described by two levels of an explanatory variable, while the latter describes the ratio of the observed number of cases to the expected number of cases, and therefore, must not be used interchangeably.^5^

## Publication bias is a crucial indicator of meta-analysis in clinical research

It is also significant that the study did not evaluate the presence of publication bias in its included studies. Despite its absence being a legitimate threat to the validity of any meta-analysis study, few meta-analysis studies include it in their analysis, even though methods to assess it has been addressed in previous studies, elaborating on its importance.^6–8^

## Estimated variation of heterogeneity between studies

Another point of consideration would be the test of between-study heterogeneity. The authors have used the Higgins I-squared statistic to analyse the meta-analysis data. However, the I-squared statistic may not be sufficiently informative as it does not consider the threshold effect. We want to suggest that Kendall’s Tau-squared statistic to be included. This Tau-squared statistic serves as the estimated variation of heterogeneity between the effects for test-accuracy observed between studies, while also considering the threshold effect.^9^

These issues are not only relevant to Gilbert et al.’s study, but also for all systematic reviews and meta-analysis which may seek to follow up on similar lines of study.
